# Effect of Exogenous Progesterone on Fetal Nuchal Translucency: An Observational Study

**DOI:** 10.7759/cureus.33023

**Published:** 2022-12-27

**Authors:** Nilajkumar D Bagde, Madhuri Bagde, Zameer Lone, Sarita Agrawal, Prasanta Nayak, Saroj K Pati

**Affiliations:** 1 Obstetrics and Gynaecology, All India Institute of Medical Sciences, Raipur, IND; 2 Obstetrics and Gynaecology, Raipur Institute of Medical Sciences, Raipur, IND; 3 Radiodiagnosis, All India Institute of Medical Sciences, Raipur, IND

**Keywords:** pharmacology, crown rump length, progesterone, first trimester ultrasound, fetal structural defects, ultrasound (u/s), pregnancy, congenital anomalies, progesterone supplement, nuchal translucency

## Abstract

Introduction

Nuchal translucency is a reliable first trimester screening test for fetal structural and chromosomal defects. Neonates with increased nuchal thickness are at greater risk for anomalies. Exogenous progesterone supplementation may affect nuchal translucency and thus the first trimester anomaly screening. We aimed to study if there was a difference in nuchal thickness between women receiving progesterone in the first trimester compared to those who were not supplemented with progesterone.

Material and methods

Forty-seven women with documented progesterone intake in the first trimester for at least 10 continuous days before the day of the nuchal translucency scan served as the study group compared to 47 other women who did not receive progesterone. Nuchal translucency was measured between 11 and 13 weeks and six days of gestation.

Results

The mean nuchal translucency increased with increasing gestation in both groups. Maximum mean nuchal translucency was greatest in the age group 18-20 years (1.35 + 0.1 millimeters) in women receiving progesterone compared to 36-40 years (1.65 + 0.49 millimeters) in controls. The mean nuchal translucency in women receiving progesterone was 1.15 + 0.26 millimeters, and in those that did not receive progesterone, it was 1.23 + 0.35 millimeters (p = 0.314).

Conclusions

Nuchal translucency increased with increasing gestation in both groups, irrespective of progesterone supplementation. There was no significant difference in mean nuchal translucency in women supplemented with progesterone compared to those that did not receive progesterone in the first trimester.

## Introduction

Nuchal translucency (NT) is a fluid-filled subcutaneous space at the back of the fetal neck that is detected by ultrasonography in the late first and early second trimesters [1]. NT is measured at a minimum crown rump length (CRL) of 45 millimeters, corresponding to 11 weeks of gestation, and a maximum CRL of 84 millimeters, corresponding to 13 weeks and six days of gestation [2]. It increases with increased gestational age. At 11 weeks of gestation, NT lies between 1.2 and 2.1 mm, and at CRL 8 mm, NT values lie between 1.9 and 2.7 mm [3]. These values remain constant regardless of the CRL between 45 and 84 mm. NT values ³3.5 mm correspond to the 99th percentile and are considered abnormal with an increased risk of aneuploidy.

Increased fetal NT is associated with fetal abnormalities [4,5]. Nuchal translucency is a reliable first trimester screening test for trisomies 21, 18, and 13, fetal structural anomalies, and neurodevelopmental defects [6-9] detected in the second or third trimester of pregnancy. Neonates with increased nuchal translucency in the first trimester of gestation and those born with structural malformations have a poor prognosis with greater morbidity and mortality [4,9].

Progesterone, a female steroid sex hormone produced by the corpus luteum in early pregnancy, creates embryo-endometrial harmony and is crucial in maintaining pregnancy [10]. "Progestogens" are a group of molecules that include the natural female sex hormones, progesterone, and 17-hydroxyprogesterone, as well as several synthetic forms, all displaying the ability to bind progesterone receptors. Exogenous progesterone use has been found to increase the nuchal translucency of the fetus [11,12]. A few studies report the contrary [13-15]. Progesterone is being increasingly used in assisted reproductive technology (ART) cycles and threatened abortions. The role of exogenous progesterone and its effects on NT are discussed as this may affect first trimester aneuploidy screening.

In this study, the primary goal was to evaluate the effects of exogenous progesterone on NT thickness in women receiving progesterone compared to those who did not receive progesterone in the first trimester.

## Materials and methods

A case-controlled observational study was carried out at the Department of Obstetrics and Gynecology, All India Institute of Medical Sciences, Raipur, Chhattisgarh, India, to determine the relationship between exogenous progesterone intake and nuchal translucency at 11 to 13 weeks and six days of gestation. A clearance from the Institutional Ethical Committee was obtained.

The estimated sample size was 100, calculated based on the standard deviation (SD) and (d) the minimum difference detected between the control and study groups for mean NT thickness, taken from a previous study [14]. Informed consent for participation was obtained after counseling the participants in their familiar language and providing adequate information regarding the study. Additional queries, if any, were addressed. The study group included all participants with a history of progesterone intake in the first trimester (at least daily for 10 days before the day of the NT scan). Women matched for age who had no history of taking progesterone preparations in the first trimester before the NT scan served as controls.

Measurement of nuchal translucency

A transabdominal scan by a single observer was performed for the evaluation of nuchal translucency. The image was magnified so that the fetal head and thorax occupied the whole screen. A mid-sagittal image of the face was obtained, defined by the presence of the echogenic tip of the nose and the rectangular shape of the palate anteriorly, the translucent diencephalon in the center, and the nuchal membrane posteriorly. The head was in line with the spine, maintaining a neutral position. Fetal skin and amnion were carefully distinguished by waiting for spontaneous fetal movement away from the amniotic membrane; alternatively, the fetus was bounced off the amnion by asking the mother to cough and/or by tapping the maternal abdomen. The widest part of nuchal translucency was measured. The measurement was taken after ensuring that the inner border of the horizontal line of the calipers was placed on the line defining the nuchal translucency thickness-the crossbar of the caliper merged with the white line of the border, not in the nuchal fluid. An average of three measurements was taken and noted as the nuchal translucency. If the umbilical cord was present around the neck, the patient was excluded from the study.

Progesterone intake

Progesterone intake was determined from history, a review of treatment sheets, and prescriptions. Patients were recalled with the prescribed drugs, and the medication was confirmed. Those women taking oral progesterone or progestogens continuously for the last 10 days were included. Women on additional medications other than iron, folic acid, and progesterone were excluded.

**Figure 1:**
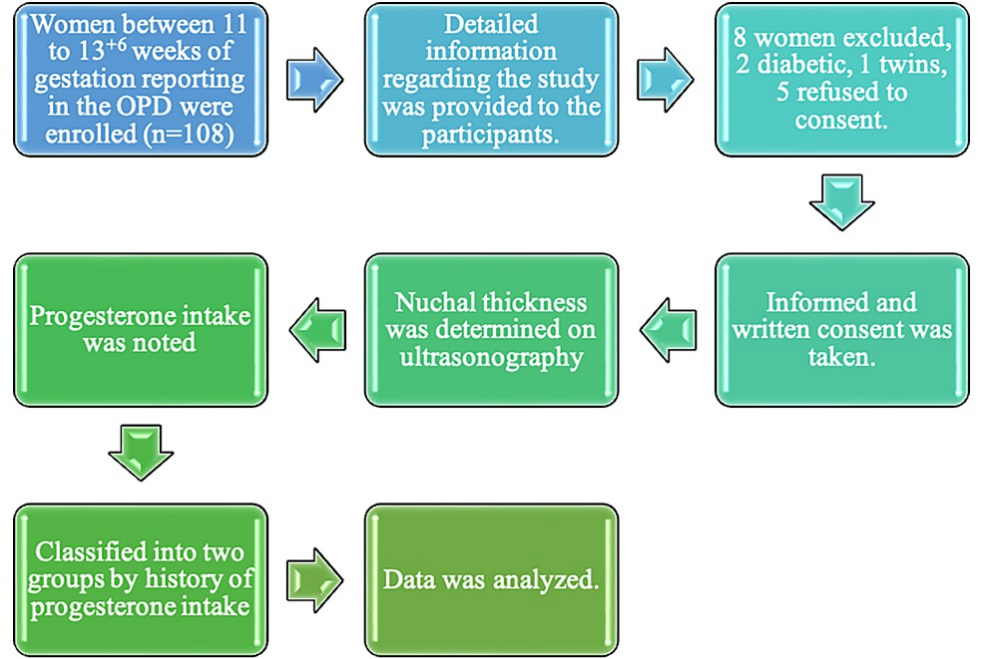
Study plan The image has been created by the authors.

Figure 1 showcases the study plan. 

Statistical analysis

Categorical variables were expressed as frequencies and compared using Pearson’s chi-squared test. Continuous variables were compared between the two groups using an independent t-test. The maternal age, gestational age, literacy, socioeconomic status, gravidity, CRL, and NT values of the progesterone and non-progesterone groups were compared. The mean NT between the progesterone and the non-progesterone groups was evaluated and also compared for the duration of progesterone intake.

IBM's statistical software, Statistical Product and Service Solutions (SPSS) 16.0 for Windows, was used for the analysis. A p-value less than 0.05 was considered statistically significant.

## Results

Most women in both groups were aged 26-30 years. There was no significant difference (p = 0.644) in the mean ages of women in the study group (27.60 years) and controls (28.02 years). There was no significant difference (p > 0.05) in literacy and socio-economic status between both groups.

Most women with a history of progesterone exposure were primigravida or gravida two, while most controls were primigravida. The mean gravidity was 2.17 in cases and 1.57 in controls. The mean parity was 0.27 for cases and 0.29 for controls and ranged from 1 to >=6 for both groups with no difference in parity between groups (p = 0.123).

The progesterone group had a mean NT of 1.15 + 0.26 mm, and the non-progesterone group had a mean NT of 1.23 + 0.35 mm. There was no significant difference (p = 0.314) in the nuchal translucency between the progesterone and non-progesterone groups.

The mean NT was highest in women receiving progesterone between the ages of 18 and 20 (Table 1), followed by those between 26 and 30 years of age. In controls, the maximum mean NT was observed in the 36-40 year age group, followed by 31-35 years. The mean NT did not differ in both groups across all age categories (p>0.05).

**Table 1:** The mean nuchal translucency at different maternal age subgroups was compared between women who received progesterone supplements (the study group) and those who did not (the control group). *millimeter; #standard deviation

Maternal age (years)	Number of subjects (N)	Nuchal translucency (mm)*		
		Study group mean + SD#	Controls mean + SD	p-value
18-20	3	1.35 ± 0	1.05 ± 0.21	0.221
21-25	20	1.03 ± 0.24	1.18 ± 0.22	0.151
26-30	46	1.24 ± 0.27	1.17 ± 0.30	0.433
31-35	19	1.08 ± 0.23	1.35 ± 0.50	0.173
36-40	3	1.10 ± 0	1.65 ± 0.49	0.221

The mean NT increased with increasing gestation in both groups (Table 2) and did not differ between the two groups at any gestation (p > 0.05). The mean NT of the progesterone group was greatest at CRL 80-84 mm (Table 3), whereas in the non-progesterone group, it was greatest at CRL 70-74.9 mm. NT did not increase uniformly with CRL, and there was no difference in NT in both groups by CRL. 

**Table 2:** The mean nuchal translucency at different gestational ages was compared between women who received progesterone supplements (the study group) and those who did not (the control group). *standard deviation

Gestational age (weeks)	Number of subjects (N)	Nuchal translucency (mm)		
		Study group mean + SD*	Controls mean + SD	p-value
11-11^+6 days^	16	1.03 ± 0.16	0.88 ± 0.19	0.145
12-12^+6 days^	40	1.11 ± 0.28	1.30 ± 0.42	0.152
13–13^+6 days^	38	1.25 ± 0.25	1.31 ± 0.24	0.377

**Table 3:** The mean nuchal translucency in different CRL subgroups was compared between women who received progesterone supplements (the study group) and those who did not (the control group). *millimeter; #standard deviation

CRL* (mm)	Nuchal translucency (mm)		
	Study group mean + SD^#^	Controls mean + SD	p-value
45-49.99	0.96 ±0.19	0.93 ± 0.27	0.765
50-54.99	1.10 ± 0.15	1.02 ± 0.19	0.403
55-59.99	0.97 ± 0.22	1.18 ± 0.39	0.332
60-64.88	1.12 ± 0.28	1.44 ± 0.45	0.091
65-69.99	1.33 ± 0.21	1.36 ± 0.28	0.823
70-74.99	1.38 ± 0.33	1.47 ± 0.50	0.593
75-79.99	1.04 ± 0.20	1.32 ± 0.21	0.146
80-84	1.50 ± 0	1.26 ± 0.137	0.137

Progesterone was administered to the majority of subjects for 30-45 days. The mean NT was 1.13 mm in women receiving progesterone therapy for < 30 days, 1.11 mm for 30-45 days, and 1.24 mm for >45 days, respectively. There was no significant difference (p > 0.05) in the nuchal translucency with respect to the duration of progesterone intake.

## Discussion

Nuchal translucency is measured at 11 to 13 + 6 weeks of gestation, corresponding to 45 to 84 mm of crown-rump length [3]. NT thickness plays an important role in determining the risk of aneuploidies [16]. The larger the NT, the higher the risk [17]. NT is a reliable first trimester screening test for fetal structural anomalies such as congenital heart defects or neurodevelopmental anomalies, which can be detected later in gestation. The prevalence of major cardiac defects, diaphragmatic hernia, exomphalos, body stalk anomaly, and fetal akinesia deformation sequence is greater in fetuses with abnormal NT compared to the general population [4].

Nuchal thickness increases with maternal age, along with the increased risk of fetal abnormalities [3,18]. Increased maternal age and NT are linked to an increased risk of Down syndrome [16].

In our study, the increase in NT thickness with increasing maternal age was not uniform. This may be due to the small sample size and the particularly small percentage of subjects who were older than 30 years of age.

The gravidity was greater in the progesterone group as compared to the non-progesterone group, similar to the results published by Kalem MN et al. [18] (p=0.019), and may have resulted from a greater frequency of progesterone prescription in women with high gravidity and a greater number of previous abortions to help maintain the current pregnancy.

The increase in mean NT with increasing gestational age and fetal CRL was not uniform in both the progesterone and non-progesterone groups in our study (Table 2). Other studies have also reported mixed results on the relation between NT and gestational age [3,11,18,19].

**Figure 2:**
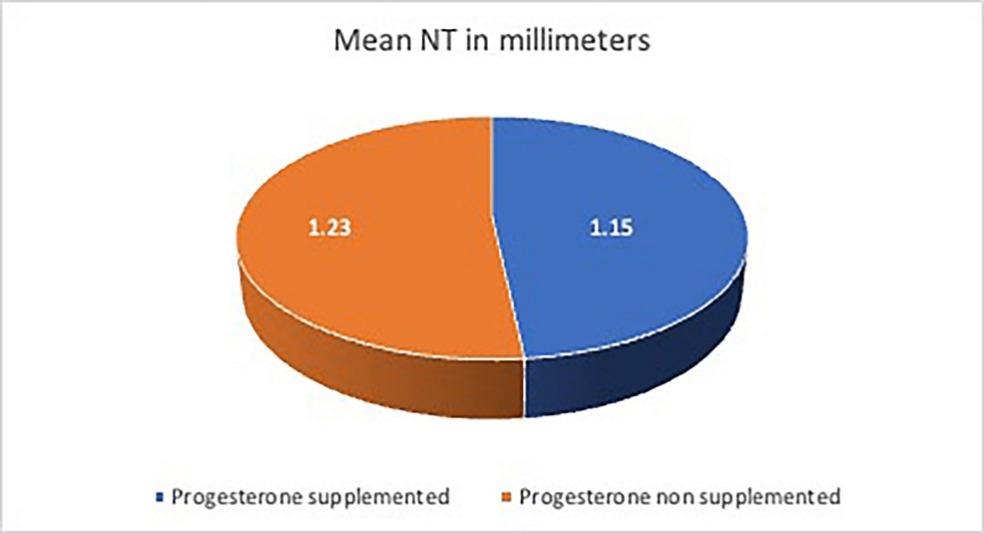
The comparison of mean NT in women supplemented with progesterone (case) and those that did not (control group) The image has been created by the authors.

On comparative analysis of mean NT in the progesterone and non-progesterone groups (Figure 2), there was no effect of exogenous progesterone on the NT thickness in our study. Comparative studies of natural and assisted conceptions did not report any difference in the NT or multiple of median (MoM) values [20,21] and one study reported a significantly lower NT in women with assisted conception compared to spontaneous pregnancies [19].

Contrasting results have been reported in other studies where NT was greater in women supplemented with progesterone [11,18,21]. In pregnancies conceived after intrauterine insemination compared to spontaneous conceptions, progesterone-supported women demonstrated a greater NT [22]. The rise in NT may have a heterogeneous etiology depending upon the underlying defect. This includes raised blood flow and consequent aberrations in fetal growth factors; cardiac failure due to congenital cardiac anomalies; amnion rupture and venous congestion in the fetal head and neck; a narrow chest or diaphragmatic hernia leading to compression of the superior mediastinum; abnormalities in the lymphatic system or lymphatic drainage; or abnormal composition of subcutaneous tissue [5].

**Figure 3:**
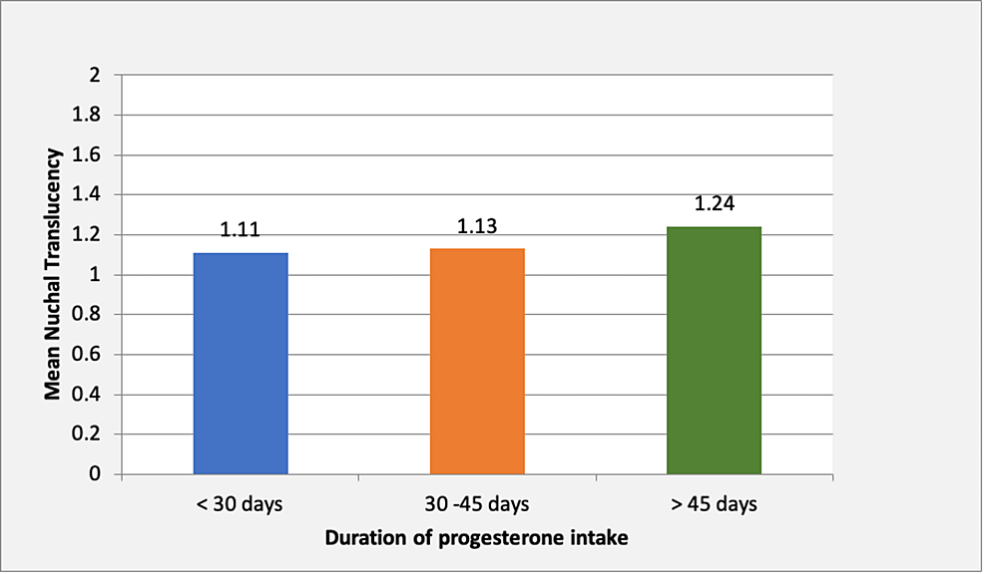
The comparison of mean NT in different subgroups based on progesterone intake duration. The image has been created by the authors.

Keçecioğlu et al. reported an increase in NT with progesterone, irrespective of the duration of exposure [21]. In our study, all participants had spontaneous pregnancies, and nuchal translucency did not differ in women who received exogenous progesterone and was also not affected by the duration of progesterone intake (p = 0.46 between the groups) (Figure 3).

## Conclusions

There is a positive correlation between nuchal translucency and gestational age. Our study revealed that nuchal translucency increases with increasing gestational age in a linear fashion, but the increase is not uniform. In this study, nuchal translucency was unrelated to maternal age. Progesterone intake did not have any effect on nuchal translucency. Women with a history of progesterone intake in the first trimester did not demonstrate greater nuchal translucencies, irrespective of the duration of exposure.
